# Evaluating the appropriateness and safety of generative AI in delivering lifestyle guidance for atrial fibrillation patients

**DOI:** 10.1038/s41598-025-34079-z

**Published:** 2025-12-29

**Authors:** Masahiro Makino, Wan Jou She, Panote Siriaraya, Satoaki Matoba, Keitaro Senoo

**Affiliations:** 1 Department of Cardiovascular Medicine, Graduate School of Medical Science, Kyoto Prefectural University of Medicine, Kyoto, Japan; 2 Faculty of Information and Human Sciences, Kyoto Institute of Technology, Kyoto, Japan; 3 Department of Cardiac Arrhythmia Research and Innovation, Graduate School of Medical Science, Kyoto Prefectural University of Medicine, Kyoto, Japan

**Keywords:** Atrial fibrillation, Lifestyle guidance, Generative AI, Large language model, Retrieval-augmented generation, Cardiology, Health care

## Abstract

Lifestyle factors play a major role in atrial fibrillation (AF) incidence, but the effectiveness of lifestyle counseling varies among individuals. Due to limited consultation time, physicians often provide only brief guidance, leaving patients to manage changes on their own. This study assessed the clinical utility of three Large Language Models (LLMs) for delivering accurate and personalized lifestyle guidance: (1) GPT-4o, (2) a retrieval-augmented model using a curated Q&A database (DB GPT), and (3) a modular RAG model retrieving evidence from PubMed (PubMed GPT). Sixty-six questions from 16 AF patients were categorized into exercise, diet, lifestyle, and other domains. Five experienced electrophysiologists independently evaluated LLM-generated lifestyle guidance and physician-provided counseling responses using ten dimensions. GPT-4o demonstrated a comparable level of scientific consensus to electrophysiologists, while achieving a lower error rate and significantly higher levels of specialized content, empathy, and helpfulness. DB GPT and PubMed GPT showed similar error rates, proportions of specialized content, empathy, and helpfulness compared to electrophysiologists, but exhibited strengths in specialized content in exercise-related and accuracy in diet-related dimensions. These findings suggest that integrating complementary model strengths may help develop safer and more reliable medical AI systems.

**Supplementary Information:**

The online version contains supplementary material available at 10.1038/s41598-025-34079-z.

## Introduction

Many studies have highlighted the importance of lifestyle changes in preventing atrial fibrillation (AF) from occurring. However, this crucial aspect of care is often overlooked due to the limited time physicians have for each patient during the outpatient clinic^1,2^.

Since the advancement of generative AI’s conversational capacity lead by ChatGPT-kind of Large Language Models (LLMs), LLMs have gained significant attention in various fields, as well as in the medical field. They demonstrate vast potentials in supporting a myriad of tasks from summarizing medical evidence to assisting diagnosis. For example, AI-based clinical decision support systems (CDSS) have been reported to be useful as diagnostic assistance tools for physicians^3,4^. In addition, research on lifestyle behavior change mediated by AI systems^5^ has shown the effectiveness of AI chatbots in facilitating behavioral changes in lifestyle, such as improved eating habits, smoking cessation, and medication adherence^6–9^. Thus, generative AI has great potential for application in the medical field, but at the same time, concerns exist regarding the appropriateness, safety, and reliability of responses due to hallucination (generation of false information)^10–12^.

This study aims to assess the appropriateness, safety, and reliability of LLM-generated lifestyle guidance for patients with AF. It will also evaluate the models’ bedside manner, empathy, and adherence to scientific consensus when presenting this guidance, and determine their overall clinical utility.

## Results

**Fig. 1:**
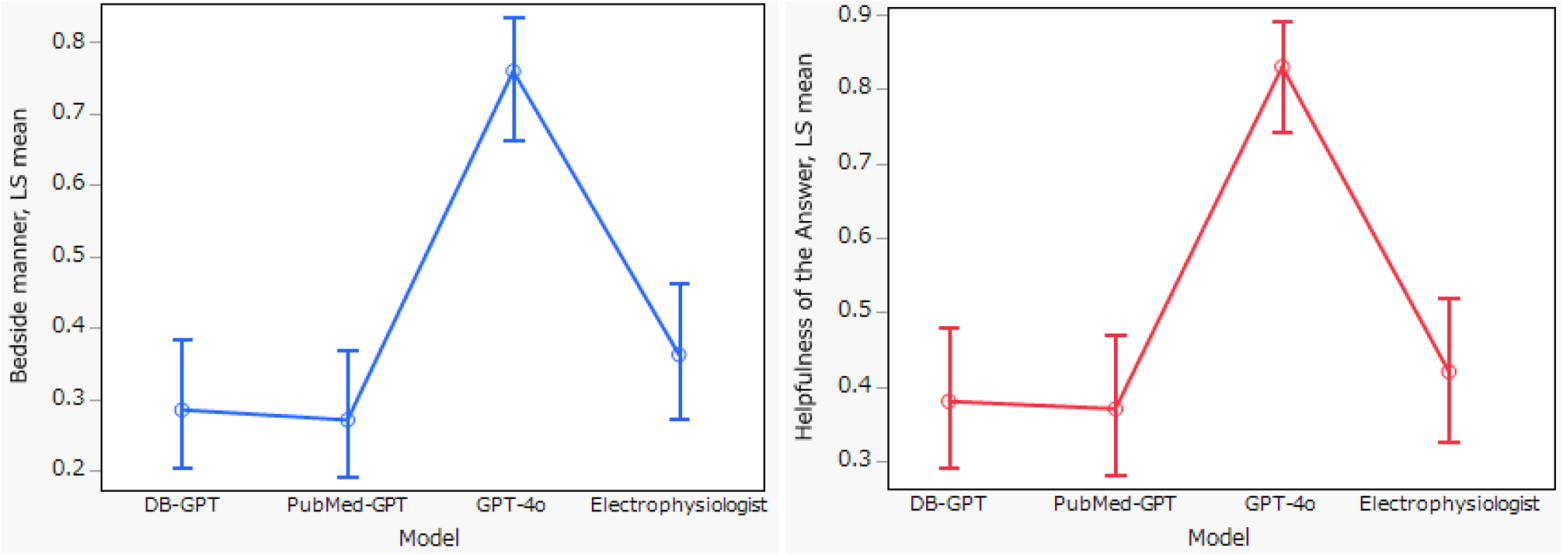
The distribution of Bedside Manner (empathy to the user) and Helpfulness across the four groups. Mean empathy scores (1 = highest, 5 = lowest) and distribution of ranking scores (1st–4th) among the four models. Lower ranking values indicate higher accuracy. Bars represent least-squares means and 95% confidence intervals obtained from the Generalized Linear Mixed Model (GLMM) analysis. For binary analysis, empathy scores of 1–2 were coded as “1” and 3–5 as “0”, while ranking scores of 1st–2nd were coded as “1” and 3rd–4th as “0”. *p* values were adjusted for multiple comparisons using Dunnett’s test with the electrophysiologist as the control.

The five evaluators were all experienced physicians, with an average of 17.2 years of clinical practice (range, 14–21 years). All evaluation dimensions were analyzed using a Generalized Linear Mixed Model (GLMM) with post-hoc multiple comparisons (Dunnett’s test, using the electrophysiologist as the control group). As shown in Table 1, Scientific Consensus was observed in 80.0% of responses for the electrophysiologist, 67.0% for DB GPT, 62.0% for PubMed GPT, and 88.0% for GPT-4o. Compared with the electrophysiologist, DB GPT (OR 0.46, 95% CI 0.20–1.05) and PubMed GPT (OR 0.35, 95% CI 0.16–0.81) showed lower odds of achieving consensus, whereas GPT-4o (OR 1.95, 95% CI 0.73–5.21) demonstrated a comparable rate. When compared with the electrophysiologist, the proportions rated as “No/mild” for Extent of Possible Harm were 75.8% for DB GPT, 78.0% for PubMed GPT, and 90.9% for GPT-4o, with no statistically significant differences observed (DB GPT: OR 0.82 [0.37–1.86]; PubMed GPT: OR 0.94 [0.41–2.14]; GPT-4o: OR 2.71 [0.98–7.51]). For Evidence of Incorrect Comprehension, GPT-4o demonstrated a significantly lower error rate compared with the electrophysiologist (OR 3.41, 95% CI 1.40–8.30), whereas DB GPT and PubMed GPT showed comparable performance (OR 1.06 [0.49–2.29] and 0.64 [0.30–1.37], respectively). Regarding Evidence of Incorrect Retrieval, GPT-4o also exhibited a significantly lower error frequency compared with the electrophysiologist (OR 3.46, 95% CI 1.28–9.31), while DB GPT and PubMed GPT showed no significant differences (both OR 0.91 [0.39–2.14]). For Evidence of Incorrect Reasoning, none of the LLMs showed statistically significant differences compared with the electrophysiologist (DB GPT: OR 0.57 [0.23–1.39]; PubMed GPT: OR 0.50 [0.20–1.22]; GPT-4o: OR 1.75 [0.62–4.92]). In Inappropriate Content, PubMed GPT demonstrated a significantly lower rate than the electrophysiologist (OR 0.32, 95% CI 0.10–0.99), while DB GPT and GPT-4o showed comparable rates (OR 0.76 [0.24–2.41] and 1.00 [0.31–3.19], respectively). For Incorrect Content, no significant differences were observed among any of the LLMs compared with the electrophysiologist (DB GPT: OR 0.67 [0.28–1.59]; PubMed GPT: OR 0.67 [0.28–1.59]; GPT-4o: OR 2.51 [0.92–6.91]). In contrast, for Specialized Content, GPT-4o produced a significantly higher proportion of responses containing specialized information than the electrophysiologist (OR 10.80, 95% CI 4.28–17.29), whereas DB GPT and PubMed GPT showed no significant differences (OR 1.45 [0.64–3.28] and 1.69 [0.75–3.79], respectively). As shown in Fig. 1, for Bedside Manner (rated on a 5-point scale, 1 = very empathetic, 5 = not empathetic), GPT-4o demonstrated significantly higher empathy compared with the electrophysiologist (odds ratio = 5.55, 95% CI 2.60–11.82), whereas DB GPT (OR = 0.70, 95% CI 0.34–1.45) and PubMed GPT (OR = 0.65, 95% CI 0.31–1.36) showed comparable levels. For Helpfulness of the Answer (rated on a 4-rank scale, 1 = best, 4 = worst), GPT-4o was rated as more helpful than the electrophysiologist (OR = 6.74, 95% CI 3.05–14.88), while DB GPT (OR = 0.85, 95% CI 0.43–1.68) and PubMed GPT (OR = 0.81, 95% CI 0.41–1.61) did not differ significantly from the electrophysiologist.

**Table 1:** Evaluation of responses from four models using the evaluation framework.

		DB GPT	PubMed GPT	GPT-4o	E.P	Odds ratio (95% CI)ª
Scientific consensus, number (%)	Yes	67 (67.0)	62 (62.0)	88 (88.0)	80 (80.0)	DB: 0.46 (0.20–1.05) PM: 0.35 (0.16–0.81) 4o: 1.95 (0.73–5.21)
	No	33 (33.0)	38 (38.0)	12 (12.0)	20 (20.0)	
Extent of possible harm, number (%)	No/mild	75 (75.8)	78 (78.0)	90 (90.9)	79 (79.0)	DB^b^: 0.82 (0.37–1.86) PM^b^: 0.94 (0.41–2.14) 4o^b^: 2.71(0.98–7.51)
	Moderate	21 (21.2)	20 (20.0)	9 (9.1)	20 (20.0)	
	Severe/fata	3 (3.0)	2 (2.0)	0 (0)	1 (1.0)	
Evidence of INCORRECT Comprehension, number (%)	Yes	33 (33.0)	42 (42.4)	15 (15.0)	34 (34.0)	DB: 1.06 (0.49–2.29) PM: 0.64 (0.30–1.37) 4o: 3.41 (1.40–8.30)
	No	67 (67.0)	57 (57.6)	85 (85.0)	66 (66.0)	
Evidence of incorrect retrieval, number (%)	Yes	29 (29.0)	29 (29.0)	12 (12.0)	27 (27.3.0)	DB: 0.91 (0.39–2.14) PM: 0.91 (0.39–2.14) 4o: 3.46 (1.28–9.31)
	No	71 (71.0)	71 (71.0)	88 (88.0)	72 (72.7)	
Evidence of incorrect reasoning, number (%)	Yes	25 (25.0)	27 (27.0)	11 (11.0)	17 (17.0)	DB: 0.57 (0.23–1.39) PM: 0.50 (0.20–1.22) 4o: 1.75 (0.62–4.92)
	No	75 (75.0)	73 (73.0)	89 (89.0)	83 (83.0)	
Inappropriate content, number (%)	Yes	16 (16.2)	24 (24.2)	14 (14.0)	14 (14.0)	DB: 0.76 (0.24–2.41) PM: 0.32 (0.10–0.99) 4o: 1.00 (0.31–3.19)
	No	83 (83.8)	75 (75,8)	86 (86.0)	86 (86.0)	
Incorrect content, number (%)	Yes	29 (29.0)	29 (29.0)	12 (12.1)	23 (23.0)	DB: 0.67 (0.28–1.59) PM: 0.67 (0.28–1.59) 4o: 2.51 (0.92–6.91)
	No	71 (71.0)	71 (71.0)	87 (87.9)	77 (77.0)	
Specialized content, number (%)	Yes	40 (40.4)	43 (43.0)	73 (73.7)	34 (34.0)	DB: 1.45 (0.64–3.28) PM: 1.69 (0.75–3.79) 4o: 10.80 (4.28–17.29)
	No	59 (59.6)	57 (57.0)	26 (26.3)	66 (66.0)	
Bedside manner (empathy to the user), mean (SD)		3.57 (1.32)	3.61 (1.38)	2.06 (1.22)	3.16 (1.22)	DB^c^: 0.70 (0.34–1.45) PM^c^: 0.65 (0.31–1.36) 4o^c^: 5.55 (2.60–11.82)
Helpfulness of the answer, mean (SD)		2.83 (1.03)	2.93 (1.11)	1.59 (0.91)	2.65 (0.88)	DB^d^: 0.85 (0.43–1.68) PM^d^: 0.81 (0.41–1.61) 4o^d^: 6.74 (3.05–14.88)

All evaluation dimensions were analyzed using a Generalized Linear Mixed Model (GLMM) with post-hoc multiple comparisons. DB: DB GPT, PM: PubMed GPT. 4o: GPT4o, E.P.: electrophysiologist. a: Dunnett’s test results with the Electrophysiologist as the reference group. b: In the analysis, moderate and severe/death categories were combined into a binary variable. c: Bedside Manner: 5-point scale (1 = very empathetic, 5 = not empathetic); scores of 1–2 were treated as “1” and 3–5 as “0”. d: Helpfulness of the Answer**:** 4-ranked scale (1 = best, 4 = worst); ranks of 1–2 were treated as “1” and 3–4 as “0”.

Subsequently, each behavioral aspect (Exercise, Diet, Lifestyle, and Other) were analyzed separately. In the Exercise category (Table 2), with the exception of the response from PubMed GPT on the “Specialized Content”, there were no significant differences observed between the responses of the three LLMs and those of the electrophysiologist. In the Diet category (Table 3), the three LLMs were evaluated to be comparable to the electrophysiologist across all dimensions. In the General Lifestyle category (Table 4), no significant differences were observed between GPT-4o and the electrophysiologist across all dimensions. The rates of agreement with Scientific Consensus for DB GPT and PubMed GPT were 56.7% and 60.0%, respectively, which were lower figures compared to the electrophysiologist’s 93.3%. The responses from these models exhibited higher rates of Incorrect Retrieval and Reasoning, as well as Inappropriate and Incorrect Content, compared with those of the electrophysiologist. In the Other category (Table 5), the percentage of Specialized Content was significantly higher for GPT-4o at 84% compared to the electrophysiologist at 48.0%. The mean score for Helpfulness of the Answer was 1.20 for GPT-4o and 2.72 for the electrophysiologist, indicating that GPT-4o was significantly more helpful. The mean score for Bedside Manner (empathy to the user) was 1.48 for GPT-4o and 3.12 for the electrophysiologist, indicating that GPT-4o had significantly higher empathy. For all other dimensions, the three LLMs were evaluated to be comparable to the electrophysiologist.

**Table 2:** Evaluation of exercise -related responses.

		DB GPT	PubMed GPT	GPT-4o	E.P	Odds ratio (95%CI)ª
Scientific consensus, number (%)	Yes	19 (76.0)	18 (72.0)	23 (92.0)	20 (80.0)	DB: 0.77 (0.13–4.40) PM: 0.60 (0.11–3.35) 4o: 3.14 (0.34–28.82)
	No	6 (24.0)	7 (28.0)	2 (8.0)	5 (20.0)	
Extent of possible harm, number (%)	No/mild	20 (80.0)	19 (76.0)	24 (96.0)	18 (72.0)	DB^b^: 1.56 (0.31–7.85) PM^b^: 1.23 (0.26–5.87) 4o^b^: 9.38 (0.64–137.62)
	Moderate	5 (20.0)	6 (24.0)	1 (4.0)	7 (28.0)	
	Severe/ fatal	0 (0)	0 (0)	0 (0)	0 (0)	
Evidence of incorrect comprehension, number (%)	Yes	5 (20.0)	10 (40.0)	2 (8.0)	10 (40.0)	DB: 2.91 (0.57–14.74) PM: 1.00 (0.23–4.31) 4o: 8.78 (1.08–71.19)
	No	20 (80.0)	15 (60.0)	23 (92.0)	15 (60.0)	
Evidence of incorrect retrieval, number (%)	Yes	3 (12.0)	5 (20.0)	1 (4.0)	7 (28.0)	DB: 3.59 (0.48–26.97) PM: 1.77 (0.28–11.14) 4o: 12.75 (0.77–211.24)
	No	22 (88.0)	20 (80.0)	24 (96.0)	18 (72.0)	
Evidence of incorrect reasoning, number (%)	Yes	3 (12.0)	5 (20.0)	2 (8.0)	6 (24.0)	DB: 2.68 (0.36–19.92) PM: 1.33 (0.21–8.33) 4o: 4.35 (0.47–40.34)
	No	22 (88.0)	20 (80.0)	23 (92.0)	19 (76.0)	
Inappropriate content, number (%)	Yes	1 (4.0)	4 (16.7)	2 (8.0)	4 (16.0)	DB: 7.59 (0.32–180.27) PM: 0.76 (0.07–8.37) 4o: 3.10 (0.22–44.00)
	No	24 (96.0)	20 (83.3)	23 (92.0)	21 (84.0)	
Incorrect content, number (%)	Yes	3 (12.0)	5 (20.0)	1 (4.0)	4 (4.0)	DB: 1.44 (0.18–11.38) PM: 0.74 (0.11–4.86) 4o: 4.98 (0.29–85.57)
	No	22 (88.0)	20 (80.0)	24 (96.0)	21 (84.0)	
specialized content, number (%)	Yes	11 (45.8)	13 (52.0)	18 (72.0)	5 (20.0)	DB: 5.78 (0.85–39.26) PM: 8.28 (1.21–56.53) 4o: 34.44 (3.85–308.18)
	No	13 (54.2)	12 (48.0)	7 (28.0)	20 (80.0)	
Bedside manner (empathy to the user), mean (SD)		3.54 (1.35)	3.20 (1.44)	1.76 (0.93)	3.24 (1.23)	DB^c^: 0.89 (0.21–3.77) PM^c^: 1.19 (0.29–4.81) 4o^c^: 13.08 (2.20–77.95)
Helpfulness of the a nswer, mean (SD)		2.76 (1.16)	2.88 (1.20)	1.60 (0.82)	2.76 (0.78)	DB^d^: 1.19 (0.29–4.78) PM^d^: 1.40 (0.35–5.59) 4o^d^: 7.11 (1.50–33.71)

All evaluation dimensions were analyzed using a Generalized Linear Mixed Model (GLMM) with post-hoc multiple comparisons. DB: DB GPT.

PM: PubMed GPT, 4o: GPT4o, E.P.: electrophysiologist. a: Dunnett’s test results with the Electrophysiologist as the reference group. b: In the analysis, moderate and severe/death categories were combined into a binary variable. c: Bedside Manner: 5-point scale (1 = very empathetic, 5 = not empathetic); scores of 1–2 were treated as “1” and 3–5 as “0”. d: Helpfulness of the Answer**:** 4-ranked scale (1 = best, 4 = worst); ranks of 1–2 were treated as “1” and 3–4 as “0”.

**Table 3:** Evaluation of diet -related responses.

		DB GPT	PubMed GPT	GPT-4o	E.P	Odds ratio (95%CI)ª
Scientific consensus, number (%)	Yes	15 (75.0)	10 (50.0)	12 (60.0)	11 (55.0)	DB: 3.05 (0.48–19.34) PM: 0.78 (0.14–4.29) 4o: 1.29 (0.23–7.26)
	No	5 (25.0)	10 (50.0)	8 (40.0)	9 (45.0)	
Extent of possible harm, number (%)	No/mild	16 (74.2)	15 (75.0)	13 (65.0)	15 (75.0)	DB^b^: 1.79 (0.25–12.84) PM^b^: 1.00 (0.17–5.86) 4o^b^: 0.61 (0.11–3.33)
	Moderate	3 (15.8)	5 (25.0)	7 (35.0)	5 (25.0)	
	Severe/ fatal	0 (0)	0 (0)	0 (0)	0 (0)	
Evidence of incorrect comprehension, number (%)	Yes	6 (30.0)	10 (52.6)	9 (45.0)	10 (50.0)	DB: 2.88 (0.48–17.29) PM: 0.82 (0.14–4.70) 4o: 1.29 (0.23–7.18)
	No	14 (70.0)	9 (47.4)	11 (55.0)	10 (50.0)	
Evidence of incorrect retrieval, number (%)	Yes	6 (30.0)	5 (25.0)	8 (40.0)	9 (45.0)	DB: 2.27 (0.38–13.68) PM: 3.05 (0.48–19.19) 4o: 1.31 (0.22–7.66)
	No	14 (70.0)	15 (75.0)	12 (60.0)	11 (55.0)	
Evidence of incorrect reasoning, number (%)	Yes	3 (15.0)	5 (25.0)	6 (30.0)	7 (35.0)	DB: 3.90 (0.48–31.39) PM: 1.84 (0.27–12.21) 4o: 1.34 (0.21–8.54)
	No	17 (85.0)	15 (75.0)	14 (70.0)	13 (65.0)	
Inappropriate CONTENT, number (%)	Yes	3 (15.8)	5 (25.0)	5 (25.0)	4 (20.0)	DB: 1.53 (0.11–21.46) PM: 0.57 (0.43–7.46) 4o: 0.57 (0.43–7.46)
	No	16 (84.2)	15 (75.0)	15 (75.0)	16 (80.0)	
Incorrect content, number (%)	Yes	6 (30.0)	8 (40.0)	8 (42.1)	9 (45.0)	DB: 2.20 (0.38–12.92) PM: 1.29 (0.23–7.21) 4o: 1.16 (0.20–6.68)
	No	14 (70.0)	12 (60.0)	11 (57.9)	11 (55.0)	
Specialized content, number (%)	Yes	11 (55.0)	8 (40.0)	13 (68.4)	7 (35.0)	DB: 3.81 (0.50–29.21) PM: 1.41 (0.19–10.52) 4o: 8.25 (0.92–74.12)
	No	9 (45.0)	12 (60.0)	6 (31.6)	13 (65.0)	
Bedside Manner (empathy to the user), mean (SD)		3.05 (1.14)	3.65 (1.35)	3.15 (1.63)	3.65 (1.14)	DBc: 2.34 (0.38–14.52) PMc: 2.68 (0.38–14.52) 4oc: 3.75 (0.62–22.91)
Helpfulness of the answer, mean (SD)		2.50 (0.89)	2.75 (1.25)	2.25 (1.33)	2.50 (1.00)	DB^d^: 1.49 (0.33–6.86) PM^d^: 0.81 (0.18–3.79) 4o^d^: 1.83 (0.39–8.52)

All evaluation dimensions were analyzed using a Generalized Linear Mixed Model (GLMM) with post-hoc multiple comparisons. DB: DB GPT.

PM: PubMed GPT, 4o: GPT4o, E.P.: electrophysiologist. a: Dunnett’s test results with the Electrophysiologist as the reference group. b: In the analysis, moderate and severe/death categories were combined into a binary variable. c: Bedside Manner: 5-point scale (1 = very empathetic, 5 = not empathetic); scores of 1–2 were treated as “1” and 3–5 as “0”. d: Helpfulness of the Answer**:** 4-ranked scale (1 = best, 4 = worst); ranks of 1–2 were treated as “1” and 3–4 as “0”.

**Table 4:** Evaluation of lifestyle-related responses.

		DB GPT	PubMed GPT	GPT-4o	E.P	Odds ratio (95%CI)ª
Scientific consensus, number (%)	Yes	17 (56.7)	18 (60.0)	28 (93.3)	28 (93.3)	DB: 0.05 (0.01–0.43) PM: 0.06 (0.01–0.52) 4o: 1.00 (0.08–12.88)
	No	13 (43.3)	12 (40.0)	2 (6.7)	2 (6.7)	
Extent of possible harm, number (%)	No/mild	20 (66.7)	23 (76.7)	29 (96.7)	25 (83.3)	DB^b^: 0.37 (0.08–1.76) PM^b^: 0.64 (0.13–3.19) 4o^b^: 6.08 (0.40–92.65)
	Moderate	8 (26.7)	6 (20.0)	1 (3.3)	5 (16.7)	
	Severe/ fatal	2 (6.6)	1 (3.3)	0 (0)	0 (0)	
Evidence of incorrect comprehension, number (%)	Yes	13 (43.3)	12 (40.0)	3 (10.0)	8 (26.7)	DB: 0.35 (0.07–1.71) PM: 0.43 (0.09–2.08) 4o: 4.05 (0.61–27.09)
	No	17 (56.7)	18 (60.0)	27 (90.0)	22 (73.3)	
Evidence of incorrect retrieval, number (%)	Yes	13 (43.3)	11 (36.7)	3 (10.0)	5 (17.2)	DB: 0.13 (0.02–0.92) PM: 0.22 (0.03–1.49) 4o: 2.69 (0.30–23.96)
	No	17 (56.7)	19 (63.3)	27 (90.0)	24 (82.8)	
Evidence of incorrect reasoning, number (%)	Yes	12 (40.0)	10 (33.3)	2 (6.7)	1 (3.3)	DB: 0.03 (0.00–0.39) PM: 0.04 (0.00–0.59) 4o: 0.46 (0.02–8.99)
	No	18 (60.0)	20 (66.7)	28 (93.3)	29 (96.7)	
Inappropriate content, number (%)	Yes	7 (23.3)	9 (30.0)	6 (20.0)	2 (6.7)	DB: 0.09 (0.01–1.18) PM: 0.04 (0.00–0.60) 4o: 0.13 (0.01–1.71)
	No	23 (76.7)	21 (70.0)	24 (80.0)	28 (93.3)	
Incorrect content, number (%)	Yes	13 (43.3)	10 (33.3)	2 (6.7)	3 (10.0)	DB: 0.05 (0.01–0.44) PM: 0.10 (0.01–0.90) 4o: 1.72 (0.14–20.74)
	No	17 (56.7)	20 (66.7)	28 (93.3)	27 (90.0)	
Specialized content, number (%)	Yes	10 (33.3)	10 (33.3)	21 (70.0)	10 (33.3)	DB: 1.00 (0.22–4.56) PM: 1.00 (0.22–4.56) 4o: 10.58 (1.82–61.58)
	No	20 (66.7)	20 (66.7)	9 (30.0)	20 (66.7)	
Bedside manner (empathy to the user), mean (SD)		3.77 (1.43)	3.87 (1.30)	2.07 (1.02)	2.80 (1.16)	DBc: 0.48 (0.26–1.79) PMc: 0.20 (0.04–0.96) 4oc: 3.27 (0.86–12.44)
Helpfulness of the answer, mean (SD)		2.87 (1.07)	3.07 (1.05)	1.47 (0.68)	2.60 (0.93)	DB^d^: 0.76 (0.21–2.69) PM^d^: 0.56 (0.15–2.06) 4o^d^: 11.77 (2.14–64.72)

All evaluation dimensions were analyzed using a Generalized Linear Mixed Model (GLMM) with post-hoc multiple comparisons. DB: DB GPT, PM: PubMed GPT, 4o: GPT4o, E.P.: electrophysiologist. a: Dunnett’s test results with the Electrophysiologist as the reference group. b: In the analysis, moderate and severe/death categories were combined into a binary variable. c: Bedside Manner: 5-point scale (1 = very empathetic, 5 = not empathetic); scores of 1–2 were treated as “1” and 3–5 as “0”. d: Helpfulness of the Answer**:** 4-ranked scale (1 = best, 4 = worst); ranks of 1–2 were treated as “1” and 3–4 as “0”.

**Table 5:** Evaluation of other responses.

		DB GPT	PubMed GPT	GPT-4o	E.P	Odds ratio (95%CI)ª
Scientific consensus, number (%)	Yes	16 (64.0)	16 (64.0)	25 (100)	21 (84.0)	DB: 0.31 (0.06–1.73) PM: 0.31 (0.06–1.73 4o: not estimable
	No	9 (36.0)	9 (36.0)	0 (0)	4 (16.0)	
Extent of possible harm, number (%)	No/mild	19 (76.0)	21 (84.0)	24 (100)	21 (84.0)	DB^b^: 0.60 (0.11–3.42) PM^b^: 1.00 (0.16–6.43) 4o^b^: not estimable
	Moderate	5 (20.0)	3 (12.0)	0 (0)	3 (12.0)	
	Severe/ fatal	1 (4.0)	1 (4.0)	0 (0)	1 (4.0)	
Evidence of incorrect comprehension, number (%)	Yes	9 (36.0)	10 (40.0)	1 (4.0)	6 (24.0)	DB: 0.48 (0.09–2.63) PM: 0.39 (0.07–2.10) 4o: 9.27 (0.57–151.30)
	No	16 (64.0)	15 (60.0)	24 (96.0)	19 (76.0)	
Evidence of incorrect retrieval, number (%)	Yes	7 (28.0)	8 (32.0)	0 (0)	6 (24.0)	DB: 0.80 (0.17–3.91) PM: 0.66 (0.43–3.13) 4o: not estimable
	No	18 (72.0)	17 (68.0)	25 (100)	19 (76.0)	
Evidence of incorrect reasoning, number (%)	Yes	7 (28.0)	7 (28.0)	1 (4.0)	3 (12.0)	DB: 0.30 (0.04–2.09) PM: 0.30 (0.04–2.09) 4o: 3.50 (0.19–64.35)
	No	18 (72.0)	18 (72.0)	24 (96.0)	22 (88.0)	
Inappropriate content, number (%)	Yes	5 (20.0)	6 (24.0)	1 (4.0)	4 (16.0)	DB: 0.67 (0.08–5.86) PM: 0.45 (0.05–3.96) 4o: 6.36 (0.31–129.57)
	No	18 (76.7)	21 (70.0)	24 (80.0)	28 (93.3)	
Incorrect content, number (%)	Yes	7 (28.0)	6 (24.0)	1 (4.0)	7 (28.0)	DB: 1.00 (0.18–5.56) PM: 1.30 (0.23–7.41) 4o: 11.97 (0.74–192.90)
	No	18 (72.0)	19 (72.0)	24 (96.0)	18 (72.0)	
Specialized content, number (%)	Yes	8 (32.0)	12 (48.0)	21 (84.0)	12 (48.0)	DB: 0.40 (0.08–2.11) PM: 1.00 (0.21–4.75) 4o: 8.30 (1.33–51.71)
	No	17 (68.0)	13 (52.0)	4 (16.0)	13 (52.0)	
Bedside manner (empathy to the user), mean (SD)		3.76 (1.23)	3.68 (1.41)	1.48 (0.65)	3.12 (1.30)	DB^c^: 0.39 (0.09–1.77) PM^c^: 0.49 (0.11–2.11) 4o^c^: 15.41 (2.00–118.94)
Helpfulness of the answer, mean (SD)		3.12 (0.88)	2.96 (1.02)	1.20 (0.50)	2.72 (0.84)	DB^d^: 0.40 (0.09–1.78) PM^d^: 0.72 (0.18–2.90) 4o^d^: 30.54 (2.17–430.35)

All evaluation dimensions were analyzed using a Generalized Linear Mixed Model (GLMM) with post-hoc multiple comparisons. DB: DB GPT.

PM: PubMed GPT, 4o: GPT4o, *E.P*.: electrophysiologist. a: Dunnett’s test results with the Electrophysiologist as the reference group. b: In the analysis, moderate and severe/death categories were combined into a binary variable. c: Bedside Manner: 5-point scale (1 = very empathetic, 5 = not empathetic); scores of 1–2 were treated as “1” and 3–5 as “0”. d: Helpfulness of the Answer**:** 4-ranked scale (1 = best, 4 = worst); ranks of 1–2 were treated as “1” and 3–4 as “0”.

## Discussion

The incidence and recurrence of AF are strongly influenced by lifestyle factors, including alcohol consumption, blood pressure control, exercise habits, diet, and obesity. Higher alcohol intake has been linked to increased recurrence after ablation, whereas even modest reductions in consumption can lower recurrence risk^13,14^. Blood pressure control is also important, as antihypertensive treatment reduces cardiovascular events in AF patients^15^, and non-dipper hypertension is associated with higher recurrence risk after ablation^16^. With regard to physical activity, regular aerobic exercise can improve AF-related symptoms, quality of life, and exercise capacity^17^. Analyses from the TRIM-AF trial indicated that reduced physical activity in patients with CIEDs was associated with increased AF burden^18^. Similarly, findings from the PREDIMED-Plus trial showed that a weight-loss-focused dietary and lifestyle intervention led to decreases in NT-pro BNP and hs-CRP levels, highlighting the importance of proactive lifestyle interventions^19^. Additionally, obesity (BMI ≥ 30 kg/m^2^) and overweight (BMI > 25 kg/m^2^) are associated with higher recurrence rates after ablation, with each 5 kg/m^2^ increase in BMI raising recurrence risk by 13%^20^. In this way, lifestyle modification plays a crucial role in AF management, underscoring the importance of tools to assess and guide such interventions.

In recent years, research has surged in using generative AI, such as ChatGPT, to coach people toward healthier lifestyle habits^21,22^. However, while they often perform well in offering general lifestyle advice, their accuracy can vary when applied to disease-specific contexts^23–25^. To address this limitation, we developed RAG-AI, which integrates specialized databases, and evaluated its accuracy in generating responses to lifestyle-related questions for AF patients. More recently, the usefulness of combining generative AI with RAG models has been demonstrated in multiple fields. For example, Mashatian et al. reported that a RAG-AI model incorporating GPT-4 and Pinecone achieved 98% accuracy in answering questions on diabetes and diabetic foot care^26^. Malik et al. also found that GPT-4o-RAG outperformed GPT-3.5 and GPT-4o alone in providing accurate recommendations for anticoagulation management during endoscopic procedures^27^. Similarly, in the field of nutrition, integrating zero-shot learning with RAG improved accuracy by 6% in extracting information on malnutrition in the elderly^28^. In the present study, our RAG-AI (DB-GPT model and PubMed GPT model) achieved accuracy comparable to electrophysiologist’s responses, particularly in the “exercise” and “diet” categories. This suggests that the use of specialized databases can provide reliable information and reduce hallucinations. However, the RAG models showed weaker performance than GPT-4o alone in areas requiring comprehensive understanding and reasoning. According to Tang et al., hallucinations in large language models can be broadly classified into misinterpretation errors, fabricated errors, and attribute errors^12^. In our evaluation, several patterns were consistent with this framework. For example, PubMed GPT occasionally produced answers that deviated from the original intent of the question (see Appendix 1), which corresponds to a misinterpretation error. Similarly, ChatGPT sometimes generated highly empathetic responses even when no direct evidence existed (see Appendix 2), which can be interpreted as an attribute error. These findings highlight differences between models: RAG-AI is designed to avoid hallucinations by restricting answers to cited guideline articles and PubMed abstracts, whereas ChatGPT, by searching the broader Internet, may generate persuasive but less evidence-grounded responses.

To address these issues, we propose enhancing RAG-AI with fact-checking AI. This approach has been shown to be effective in other domains; for instance, incorporating explainable fact-checking AI into x-ray report generation improved accuracy by more than 40%^29^. Applying similar strategies could enhance the reliability of RAG-AI responses, reduce hallucinations, and broaden its applicability in clinical question answering.

### Limitation

The clinical application of this study has several limitations. First, the PubMed GPT processes Japanese questions by translating them into English, extracting keywords, and citing relevant PubMed abstracts. This workflow may alter the intent of the original question, and inadequate keyword extraction can lead to errors in cited information, increasing the risk of LLM-specific hallucinations. Previous studies using Chat GPT reported reference-based accuracy as low as 7% and about 20% for medical questions^30,31^, suggesting that translation and keyword selection errors could affect results. Second, Chat GPT responses were obtained in Japanese, and LLM accuracy in non-English languages is reported to be about 10% lower than in English^31^. The RAG-AIs generated responses in English, which were then translated to Japanese via DeepL. Therefore, results might differ if all responses were originally in English. Accuracy could be improved through optimized prompts and multi-step validation. Third, although GPT-4o was used, results may vary with other chatbots or future versions. Reports indicate GPT-4o outperforms GPT-3.5^27^, but overall response accuracy remains suboptimal according to Li et al.’ report^32^, consistent with our findings. Fourth, 66 questions were collected from AF patients, but they may not cover all potential queries, and editing questions could improve AI accuracy. Fifth, responses were obtained only once per question. While reproducibility may vary, existing research suggests single-response evaluation is sufficient, though future studies should verify consistency to improve reliability. Lastly, all evaluations were performed by electrophysiologists. Although their expertise in specific areas such as exercise and nutrition was limited, they routinely provide lifestyle guidance to AF patients and therefore possess sufficient knowledge regarding lifestyle counseling for AF patients. As such, the variability in evaluation results is expected to be limited. However, expanding the number and diversity of evaluators, along with prompt optimization, will be important for developing more accurate and safer medical AI systems.

In conclusion, original GPT-4o was a balanced model in providing medical information, showing high reasoning ability and empathy. On the other hand, RAG-AI (DB GPT and PubMed GPT) suppressed hallucination and was confirmed to provide highly accurate answers, especially in the areas of exercise and diet. Combining the advanced reasoning ability and empathy of GPT-4o with the hallucination suppression function of DB GPT and PubMed GPT, which utilize RAG, may enable the construction of more accurate and safer medical AI systems. In particular, the use of RAG models would be effective in areas where specialized knowledge is required, and the strengths of GPT-4o would be demonstrated in situations where more flexible and comprehensive information provision is required.

## Method

This observational study was conducted at a single institution (Kyoto Prefectural University of Medicine Hospital). The study protocol was approved by the Ethics Committee of the institution (IRB-ERB-C-3102) and carried out in accordance with the principles outlined in the Declaration of Helsinki.

### Implementation of the LLM for medical question and answering

When examining the use of LLM for medical question and answering tasks, three different approaches were examined in this study. This includes 1) direct prompting using a state-of-the-art general purpose large language model (GPT-4o model), 2) a retrieval augmented generation (RAG) approach which retrieves external medical knowledge from a database of question and answers curated by the authors (DB-GPT model) and 3) a three-stage modular RAG approach which analyses and summarizes external medical knowledge from relevant academic papers indexed in the PubMed database to provide responses that are better grounded in academic literature (PubMed GPT model).

### Baseline model: GPT-4o

The answers in the “GPT-4o” condition were generated using the latest GPT model available at the time of the study, namely the GPT-4o model, developed by OpenAI^33^. This model has shown strong performance in medical question and answering tasks^34^, even achieving an overall correct response rate of 93.2% on the 2024 Japanese Medical Licensing Exam^35^ without prompt engineering. In this study, the model was prompted with a simple instruction, aimed at setting the role of the model as a doctor offering medical advice (e.g. “You are a doctor offering advice. Please answer the following questions”) and was used to provide answers to the medical questions.

### RAG based on a curated Q&A knowledge base: (DB-GPT)

While traditional large language models have demonstrated strong performance, they still face critical challenges, particularly in a domain as sensitive as healthcare. A key issue concerns their tendency to hallucinate information, generating responses which at a first glance may seem plausible, but are factually incorrect thus potentially causing harm or undermining trust^36^. Retrieval Augmented Generation (RAG), refers to a mechanism in which the model enhances its response by retrieving information from an external knowledge base, one which is timely and carefully verified. Such an approach has been shown to produce responses which are less prone to hallucinations^36^. The model used in the “DB-GPT” condition adopted this approach. More specifically, the model was programmed to retrieve information from a local FAQ-style database to help answer user questions. The database consists of a collection of question–answer pairs that was constructed based on Review article “Lifestyle and Risk Factor Modification for Reduction of Atrial Fibrillation: A Scientific Statement from the American Heart Association”^37^. To retrieve relevant information, the questions from the database and the user query were vectorized into embeddings using the all-MiniLM-L6-v2 model. The similarity between the user query vector and the vectors of each question was compared using their dot product, and the 6 most relevant question and answer pairs (determined by those which showed the highest degree of similarity to the user query) were selected and then combined into a single prompt for GPT-4. The GPT-4 model was asked to generate a synthesized answer based only on the retrieved text. If the question cannot be addressed by the data, the model was instructed to clearly indicate that it was not able to provide an answer. More specifically, the following prompt was used: (System: “Synthesize a comprehensive answer from the following q&a dataset for the given question. Always use the provided text to support your argument. If the question cannot be answered in the document, say you cannot answer it”) and (assistant: “dataset: {DocumentList}, Question: {question}, Answer:”), with the “DocumentList” representing the relevant Question and Answer pair retrieved from the database and “question” representing the current user question.

### RAG based on academic literature knowledge base: PubMed GPT

 For the “PubMed GPT” model, we adopted an Advanced/Modular RAG approach. This involves decomposing the RAG system into smaller modules which specialize on specific tasks^38,39^. Previous studies have shown that such a multi-layer approach could perform particularly well in medical Q&A tasks^40^. In our case, we adopted a three-layer approach, consisting of a “pre-retrieval”, “post-retrieval” and “generation” process^40^. When processing Japanese questions, the PubMed GPT first translates them into English, extracts relevant keywords, and cites pertinent PubMed abstracts. In the first “pre-retrieval” stage, a GPT-4 model was used to construct and refine a query (using 10 few-shot examples) to search for relevant literature on the PubMed Database. The query was then used to retrieve the 50 most relevant research papers from the PubMed database through the Entrez API. In the following “post-retrieval stage”, a selection process was carried out, in which another GPT-4 model was used retain only the papers which likely contained answers to the user question based on the title and abstract of the papers. In the final “generation” stage, the original user question, along with the title and abstract of the related papers selected from the second stage were provided as an input to another GPT-4 model which was tasked with using the selected abstracts to generate an answer to that question.

### Development of a lifestyle questionnaire

**Fig. 2:**
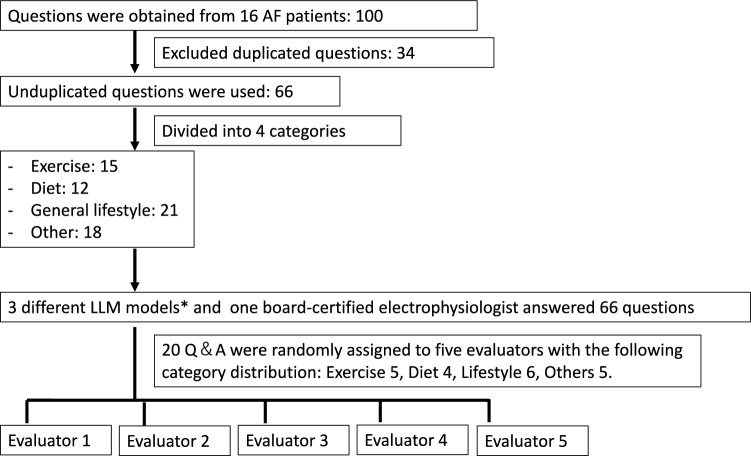
Workflow for preparing and assigning lifestyle questions to evaluators. *3 different models; original Chat GPT4o, PubMed GPT and DB-GPT.

In this study, about 10 lifestyle-related questions were obtained for each of 16 AF patients, for a total of 100 responses. Among the participants, 13 (81.3%) were male, the mean age was 69.4 ± 7.5 years, and 62.5% had paroxysmal AF. Duplicate questions were eliminated from the questions obtained, resulting in a final list of 66 unduplicated questions. These questions were grouped into four categories (exercise, diet, general lifestyle, and other) (see Supplementary Table 1 for details). The 66 questions obtained were then answered using each of the three types of generative AI (original Chat GPT4o, PubMed GPT and DB-GPT). To provide a physician’s response representative of usual clinical care for comparison with the three LLM models, we obtained answers to the same 66 questions from one electrophysiologist (S.S.), who was allowed to use the Internet when formulating responses. This resulted in a total of four responses being generated for each question (Fig. 2).

### Evaluation of AI-generated medical responses and an usual clinical care (electrophysiologist’s response)

Five evaluators (T.M, Y.K, T.H, N.N, K.K), who are board-certified electrophysiologists, were sent a Google form with 20 randomly selected questions out of 66 questions and 4 response models to them. The 20 randomly selected questions were chosen with weighting based on the number of questions in each category: 5 from the exercise category, 4 from the diet category, 6 from the general lifestyle category, and 5 from the other category. Evaluation dimensions included Scientific Consensus, Extent of Possible Harm, Evidence of Incorrect Comprehension, Evidence of Incorrect Retrieval, Evidence of Incorrect Reasoning, Inappropriate Content, Incorrect Content, Specialized Content, Bedside Manner and Helpfulness of the Answer^41^. Of these, Extent of Possible Harm was rated on a 3-point scale of mild, moderate, severe or deadly, while the other measures were rated on a 2-point scale. In addition, for Helpfulness of the Answer, the responses of the four models were ranked, and Bedside Manner was rated on a 5-point scale. For example, For the evaluation of Scientific Consensus, responses from the four models, including three GPT models and one electrophysiologist, S.S., were independently evaluated by five evaluators. Five evaluators assessed all responses in a blinded manner. The source of each response (LLM or electrophysiologist) was not disclosed to the evaluators to minimize potential subjective bias and to ensure a scientifically rigorous and reliable study design. Each evaluator judged whether a response represented content that could achieve scientific consensus. The exercise category included five questions, resulting in twenty-five Yes/No evaluation, the diet category included four questions, resulting in twenty Yes/No evaluation, the lifestyle category included six questions, resulting in thirty Yes/No evaluation, and the other category included five questions, resulting in twenty-five Yes/No evaluation. In total, one hundred Yes/No evaluation were obtained for Scientific Consensus. Other dimensions, such as Incorrect Reasoning and Inappropriate Content, were evaluated in the same way.

### Statistical analysis

For each clinical question (Scientific Consensus, Extent of Possible Harm, Evidence of Incorrect Comprehension, Evidence of Incorrect Retrieval, Evidence of Incorrect Reasoning, Inappropriate Content, Incorrect Content, Specialized Content, Bedside manner and Helpfulness of the answer), four sets of responses (three LLM models and one board-certified electrophysiologist) were evaluated by five independent evaluators. All evaluation results were analyzed using a generalized linear mixed model (GLMM). The model included model type (electrophysiologist or LLM models), category of question (i.e. lifestyle, exercise, diet, others), and evaluator as fixed effects, and question ID as a random effect to account for correlations among responses to the same clinical question. For each model, odds ratios and 95% confidence intervals were estimated, and significance levels were adjusted using Dunnett’s test with the electrophysiologist as the reference group. For the GLMM analysis, the ordinal variables were dichotomized as follows; 1) For “Extent of Possible Harm” variable, the “moderate” and “severe/death” categories were combined, 2) For “Bedside Manner” variable, which was evaluated on a 5-point scale (1 = very empathetic, 5 = not empathetic), 1–2 were coded as 1 (high empathy) and 3–5 as 0 (low empathy), 3) For “Helpfulness of the Answer” variable, which was evaluated on a 4-point scale (1 = best, 4 = worst), 1–2 were coded as 1 (highly helpful) and 3–4 as 0 (less helpful). All analyses were performed using Microsoft Excel and JMP statistical software version 18.

## Supplementary Information

Supplementary Information 1.

Supplementary Information 2.

Supplementary Information 3.

## Data Availability

The data supporting the findings of this study are available upon request from the corresponding author. Due to privacy and ethical concerns, the data are not publicly available.
