# Supplementary material for: Evaluating the appropriateness and safety of generative AI in delivering lifestyle guidance for atrial fibrillation patients
Source: Sci Rep. 2025 Dec 29;16:3961. doi: 10.1038/s41598-025-34079-z (PMC12855944; doi:10.1038/s41598-025-34079-z)
Supplement: Supplementary file 1 — Supplementary Information 1. [file 41598_2025_34079_MOESM1_ESM.docx]

**Appendix 1)** Chat GPT (GPT4o’s Answer) searches for information on the Internet to generate answers, so even questions that are not directly relevant could be evaluated as highly empathetic answers by the evaluator, the electrophysiologist

| Category | Exercise |
| --- | --- |
| Question | How much exercise is recommended? Maybe a walk for 30 minutes to an hour or so. |
| GPT‑4o’s Answer | When a patient with atrial fibrillation exercises, it is important to first consider your condition and medical condition. Instead of building up muscle mass and increasing the amount of exercise, try to start with light exercise and continue to do as much exercise as you can perform without restrictions due to your medical condition. Specifically, starting with a walk is a good approach. Starting with a 15-minute walk and gradually increasing the time will help increase exercise endurance. 30-minute to one-hour walks are less demanding on the body and are suitable for improving cardio-regulatory capacity. However, it is important to always consult with a physician or appropriate health care professional to confirm the range and intensity of safe exercise before beginning any exercise alone. Also, if you experience any unusual symptoms during exercise, such as shortness of breath, excessive fatigue, or chest pain, stop exercising immediately and consult your physician. |

| Category | diet |
| --- | --- |
| Question | I drink alcohol (3 x 350 ml beer) almost every night. Is there any relationship between the amount of alcohol I drink and atrial fibrillation? How much is it safe to drink? |
| GPT‑4o’s Answer | There is a link between atrial fibrillation and alcohol consumption. Studies have shown that excessive alcohol consumption increases the risk of atrial fibrillation. Alcohol also dilates blood vessels and increases blood pressure, which can lead to the development of atrial fibrillation. The amount the patient is currently drinking (3 x 350 ml beers) is generally considered excessive alcohol consumption, which may contribute to the worsening of atrial fibrillation. The appropriate amount of alcohol is limited to about 14 units (about 1.5 liters of beer) per day for men and about 7 units (about 750 ml of beer) per day for women. However, this is a guideline for generally healthy adults and will vary depending on individual medical conditions and physical condition. Assuming you are a patient with atrial fibrillation, it is best to abstain from alcohol consumption altogether, but if this is not possible, it is recommended that you reduce the amount and frequency of alcohol consumption. Make it a few times a week rather than a nightly habit. If you still have difficulty stopping drinking, consider consulting a physician or professional counselor for assistance. Reducing alcohol consumption is very important for maintaining good health. However, it is strongly recommended that you always consult with a health care professional before taking any specific action, as this information is general advice and may vary depending on your individual situation. |

| Category | general lifestyle |
| --- | --- |
| Question | When I have an irregular heartbeat, I feel that I am better if I talk with others, but is it better to rest alone? |
| GPT‑4o’s Answer | Atrial fibrillation is a situation in which the heart's blood flow is weaker than normal as a result of parts of the heart contracting faster or more irregularly than normal. This prevents the heart from pumping blood efficiently throughout the body. High stress in this situation can exacerbate the symptoms. If you find that talking to others relaxes you and reduces your stress, then this is a healthy method of stres relief. However, if you are not feeling well or your symptoms are worsening, you should take a break. These self-care methods are only part of the story and may require treatment from a medical perspective. It is recommended that you check up with your health care provider on a regular basis and consult with them immediately if your symptoms change or worsen. |

| Category | Other |
| --- | --- |
| Question | Is it inherited? |
| GPT‑4o’s Answer | Atrial fibrillation is a complex disease that is usually caused by a combination of factors. These include age, history of heart disease, lifestyle and health conditions such as high blood pressure, diabetes, sleep apnea, and weight. Heredity is one factor in part, and studies indicate that some people may have a genetic predisposition to developing atrial fibrillation. However, since heredity is only one factor, having a parent with atrial fibrillation does not necessarily mean that the child will develop the condition. Therefore, even if you believe you have inherited atrial fibrillation, you can minimize your risk of the disease by continuing a healthy lifestyle and having regular medical follow-ups. If you have specific risks or concerns, it is recommended that you discuss these with your physician or genetic counselor. |
