# Supplementary material for: Evaluating the appropriateness and safety of generative AI in delivering lifestyle guidance for atrial fibrillation patients
Source: Sci Rep. 2025 Dec 29;16:3961. doi: 10.1038/s41598-025-34079-z (PMC12855944; doi:10.1038/s41598-025-34079-z)
Supplement: Supplementary file 2 — Supplementary Information 2. [file 41598_2025_34079_MOESM2_ESM.docx]

**Appendix 2)** Cases where the PubMed GPT generated answers that were different from the intent of the question

| Question | Answer |
| --- | --- |
| I go to a sports club, but can I learn to swim? | Apologies for the confusion, but the question is asking about personal ability to swim, which cannot be answered based on the provided abstracts. The abstracts are expected to provide information about the relationship between atrial fibrillation, physical activity, and swimming, not about individual swimming capabilities. |
| What behaviors may trigger a recurrence? | Several behavioral triggers can potentially lead to relapse in individuals with substance use disorders. Exposure to drugs themselves, drug-associated environmental stimuli, and stress can trigger drug-seeking behavior and potentially lead to relapse. For example, small "priming" doses of drug, stress, or drug-associated cues and locations can trigger cocaine seeking after a period of abstinence. Similarly, stressful events, drug-associated cues, or re-exposure to a small amount of alcohol can trigger a chain of behaviors that frequently culminates in relapse. Furthermore, exposure to drug-associated cues, stress, and 'priming' injections of the drugs themselves can trigger drug craving in human drug addicts. In the context of alcohol use disorder, environmental factors such as stress and re-exposure to alcohol can trigger relapse. Finally, psychological stress and drug availability can interact following rehabilitation, contributing to the high relapse potential. |
| What are the drawbacks of prolonged seizures? | The disadvantages of prolonged seizures include potential brain damage, increased morbidity and mortality, and post-seizure complications. According to paper, prolonged seizures can lead to brain damage regardless of whether they are generalized or focal in onset. The damage is secondary to excessive excitability, with seizures causing massive depolarization of neurons leading to excessive glutamate release, which results in increased intracellular calcium, causing a cascade of changes that ultimately result in cell death. In the paper, it is stated that status epilepticus, a condition characterized by prolonged seizures, is associated with substantial morbidity and mortality. The outcome of status epilepticus is influenced by factors such as the etiology and duration of the seizures, and the patient's age. The study suggests that bursts of seizures, which are short-term seizure clusters, are associated with poorer predictive outcomes. Other study highlights that childhood convulsive status epilepticus (CSE) is associated with substantial neurological, cognitive, and behavioral morbidity. The additional effect of CSE characteristics such as seizure duration seems to be less than previously believed, but it still contributes to the overall negative impact. The other study discusses Todd's paralysis, a temporary limb weakness or hemiplegia that typically occurs following a seizure. The duration of Todd's paralysis can range from minutes to days, depending on the type of seizure or whether the patient has experienced cortical structural damage. Finally, the research suggests that bilateral tonic-clonic seizures, a type of prolonged seizure, are a major risk factor for sudden unexpected death in epilepsy (SUDEP). The severity of respiratory dysfunction and seizure characteristics were not associated with time to initial recovery of awareness, but a shorter time to recovery of awareness was significantly associated with a younger age. |
